# Breath biomarkers in idiopathic pulmonary fibrosis: a systematic review

**DOI:** 10.1186/s12931-019-0971-8

**Published:** 2019-01-11

**Authors:** Conal Hayton, Dayle Terrington, Andrew M. Wilson, Nazia Chaudhuri, Colm Leonard, Stephen J. Fowler

**Affiliations:** 1 Division of Infection, Immunity and Respiratory Medicine, School of Biological Sciences, Faculty of Biology, Medicine and Health, The University of Manchester, Manchester, UK; 2 Norwich Medical School, University of East Anglia, Norwich, UK; 3 North West Lung Centre, Manchester University NHS Foundation Trust, Manchester, UK

**Keywords:** Idiopathic pulmonary fibrosis, Breath tests, Nitric oxide, Volatile organic compounds, Exhaled breath condensate

## Abstract

**Background:**

Exhaled biomarkers may be related to disease processes in idiopathic pulmonary fibrosis (IPF) however their clinical role remains unclear. We performed a systematic review to investigate whether breath biomarkers discriminate between patients with IPF and healthy controls. We also assessed correlation with lung function, ability to distinguish diagnostic subgroups and change in response to treatment.

**Methods:**

MEDLINE, EMBASE and Web of Science databases were searched. Study selection was limited to adults with a diagnosis of IPF as per international guidelines.

**Results:**

Of 1014 studies screened, fourteen fulfilled selection criteria and included 257 IPF patients. Twenty individual biomarkers discriminated between IPF and controls and four showed correlation with lung function. Meta-analysis of three studies indicated mean (± SD) alveolar nitric oxide (C_alv_NO) levels were significantly higher in IPF (8.5 ± 5.5 ppb) than controls (4.4 ± 2.2 ppb). Markers of oxidative stress in exhaled breath condensate, such as hydrogen peroxide and 8-isoprostane, were also discriminatory. Two breathomic studies have isolated discriminative compounds using mass spectrometry. There was a lack of studies assessing relevant treatment and none assessed differences in diagnostic subgroups.

**Conclusions:**

Evidence suggests C_alv_NO is higher in IPF, although studies were limited by small sample size. Further breathomic work may identify biomarkers with diagnostic and prognostic potential.

**Electronic supplementary material:**

The online version of this article (10.1186/s12931-019-0971-8) contains supplementary material, which is available to authorized users.

## Introduction

Idiopathic pulmonary fibrosis (IPF) is the most common form of interstitial lung disease (ILD) with an estimated incidence of 2.8–9.3 per 100,000 per year in Europe and North America [1]. It is associated with high morbidity and mortality with a reported median survival of approximately three years [2]. Challenges exist, particularly in relation to diagnosis and clinical phenotyping [3, 4]. Diagnostic criteria for IPF have evolved over time and have recently been updated [5]. There is risk of misdiagnosis, particularly as inter-observer agreement amongst clinicians is variable [6, 7]. Surgical biopsy rates remain low due to concerns regarding associated mortality and morbidity, particularly in an elderly, comorbid patient group [8]. In addition, it is well recognised that significant heterogeneity exists within the IPF population in relation to disease progression [9, 10]. At present, lung function parameters remain the most widely used tool to monitor disease activity in IPF and the most commonly used end point in therapeutic trials [11]. An absolute annual decline in forced vital capacity (FVC) of 10% or diffusion capacity for carbon monoxide (D_LCO_) of 15% has been shown to be an accurate predictor of mortality in IPF [12–14]. However, baseline FVC measurements do not have strong prognostic value and limitations exist with respect to interpretation of longitudinal changes [15, 16]. Baseline D_LCO_ may be slightly better at predicting mortality in IPF [2], however results may be significantly affected by the presence of co-morbidity, such as pulmonary hypertension and emphysema [17]. Other physiological parameters associated with disease prognosis include total lung capacity (TLC) and 6-min walk distance [14, 18]. Composite scoring systems such as the Composite-Physiologic Index (CPI) and Gender Age Physiology (GAP) index [19, 20], which incorporate demographic and physiological data, may represent more accurate prognostic models.

It is recognised that there is a need for additional biomarkers to augment the diagnostic process, facilitate clinical phenotyping, improve accurate disease monitoring and identify potential therapeutic targets in IPF [21, 22].

Several serological markers, including Krebs von den Lungen-6 (KL-6), Surfactant protein-A (SP-A) and D (SP-D), and matrix metalloproteinase-7 (MMP-7) have been identified as having potential diagnostic and prognostic value [23–28], while the PROFILE (Prospective Observation of Fibrosis in the Lung Clinical Endpoints) study, an ongoing prospective multicentre study aimed at biomarker discovery, has identified further novel serological protein markers with prognostic potential [29, 30].

**Fig. 1:**
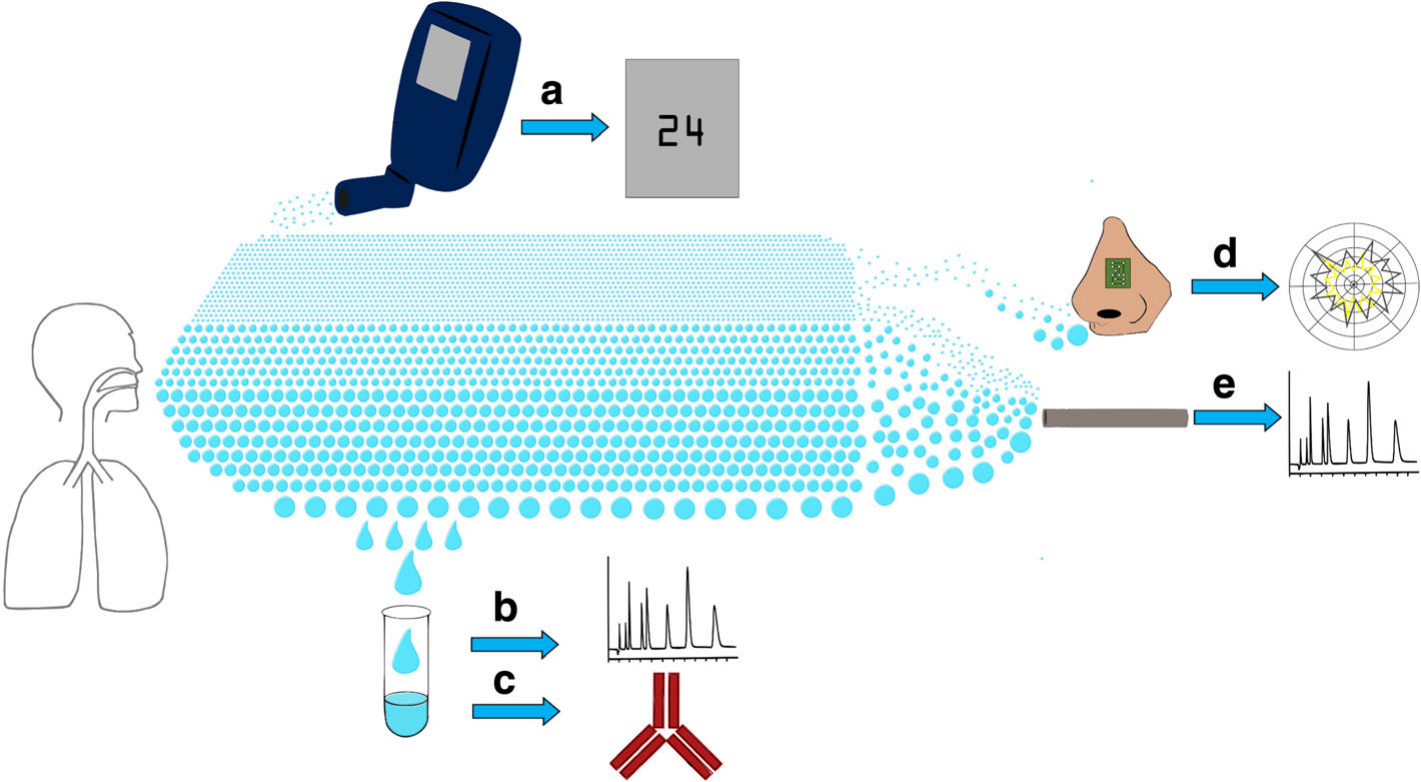
The spectrum of breath analysis. Exhaled breath contains a vast quantity of molecules and particles, ranging in size and volatility, which can be captured using analytical techniques. Nitric oxide is a very small, volatile compound which can be detected using an online analyser which will provide an instant measurement of the concentration in exhaled breath (**a**). Large, non-volatile particles can be detected in exhaled breath condensate, either through liquid chromatography-mass spectrometry (**b**) or enzyme immunoassay (**c**). Volatile organic compounds (VOCs) of varying size can be detected by using various technologies including electronic noses (**d**) or gas-chromatography mass spectrometry (**e**)

Exhaled breath may represent an alternative source of novel biomarkers in IPF. There has been an interest in breath research in respiratory disease for many years [31]. In asthma, exhaled nitric oxide (NO) has successfully transitioned from research to clinical practice and its use is now recommended in international clinical guidelines for diagnosis and monitoring [32–35]. Fractionated exhaled nitric oxide measured at a flow rate of 50 ml/s (FeNO_50_) using either a chemiluminescence, electrochemical or laser analyser is used as a surrogate marker of inflammation in the airways [33]. Capturing FeNO at different flow rates also allows estimation of the concentration of NO at the level of the alveolus (C_alv_NO) and airway wall (C_aw_NO), as well as diffusion capacity (D_aw_NO) and airway flux (J_aw_NO) [36, 37]. An alternative medium for breath sampling is exhaled breath condensate (EBC), which is comprised of condensed water vapour with small amounts of non-volatile and water-soluble volatile molecules within [38, 39]. Following collection, relevant analytes can be measured using specific assays or mass spectrometry [39]. More recently, interest in volatile organic compounds (VOCs) as a potential source of biomarkers in respiratory disease has been gathering pace [40–43]. In particular high-throughput “breathomics” using gas chromatography-mass spectrometry (GC-MS), may allow discovery of novel volatile compounds with highly specific diagnostic, therapeutic or prognostic potential [40, 42]. These methods have been used to identify VOCs in a number of respiratory diseases including asthma [44], COPD [45], pneumonia [46] and lung cancer [47]. Electronic noses (“E-noses”), sensors which detect specific patterns of VOCs, have also been tested in a number of respiratory conditions and may represent a more clinically accessible diagnostic tool [48]. Figure 1 details the breadth of current approaches for breath analysis.

Breath sampling represents an attractive investigative method, being both non-invasive, simple to collect and unlimited in quantity. However, the utility of breath analysis in IPF is unclear. The aim of this systematic review was to investigate the role of breath biomarkers in IPF. The primary aim was to establish which breath biomarkers are significantly different in the IPF population compared to healthy controls. We also hoped to answer whether biomarkers correlate with lung function parameters within the IPF population, differ between treated and untreated patients and between patients with and without honeycombing.

## Materials and methods

We performed this systematic review using PRISMA (Preferred Reporting Items for Systematic Reviews and Meta-Analyses) guidelines [49]. The review was registered with the International prospective register of systematic reviews (PROSPERO) (Registration ID CRD42017078645).

### Search strategy and study selection

Two reviewers performed independent electronic database searches in January 2018. Databases searched were MEDLINE (using both PubMed and Ovid), EMBASE and Web of Science. Searches were performed using a combination of MeSH and key word terms. The search terms used were (idiopathic pulmonary fibrosis OR pulmonary fibrosis OR lung diseases, interstitial OR fibrosing alveolitis OR diffuse parenchymal lung disease OR usual interstitial pneumonia) AND (breath tests OR volatile organic compounds OR metabolomics OR breath analysis OR breath biomarkers OR breathomics OR exhaled breath).

Study selection was performed based on the following eligibility criteria. Inclusion criteria were: age over 18; a diagnosis of idiopathic pulmonary fibrosis based on international guidelines[2, 50]; primary research involving breath biomarkers; comparison control group of healthy participants or comparison within IPF population based on lung function or treatment strategy. Exclusion criteria were: non-human studies; review articles; research in abstract form only; non-english language publications. Each reviewer performed abstract and full text review to identify studies which met selection criteria. Reference lists of retrieved articles were reviewed to identify additional papers. Google Scholar (https://scholar.google.co.uk) was used to identify any additional articles which may have cited the retrieved articles.

### Data extraction and quality assessment

Data extraction and quality assessment of all the selected studies was performed independently by both reviewers. Quality was assessed using the QUADAS-2 tool [51]. This facilitates the assessment of studies of diagnostic tests for bias and applicability in four domains; patient selection, index test, reference test and flow and timing. In this review, the index test was the biomarker under investigation and the reference test was a diagnosis of IPF based on international guidelines [2, 50].

Data extraction was performed using an electronic data collection form. Data collected included country and year of publication, method of breath collection and analytical technique, number of IPF and control patients, baseline characteristics (e.g. age, gender, smoking status) and significant differences between groups, treatment regimens, reported lung function parameters, biomarker levels and reported differences between groups.

All studies identified were included in qualitative data synthesis. Quantitative data synthesis was performed where three or more studies reported the same outcome. This consisted of random effects meta-analysis of mean difference in biomarker levels (IPF vs controls) with I^2^ statistic to assess heterogeneity. Reported mean and standard deviation (SD) of biomarker levels were included in the analysis. If studies reported standard error (SE), this was converted to SD. Statistical analysis was performed using OpenMeta-Analyst software [52].

## Results

**Fig. 2:**
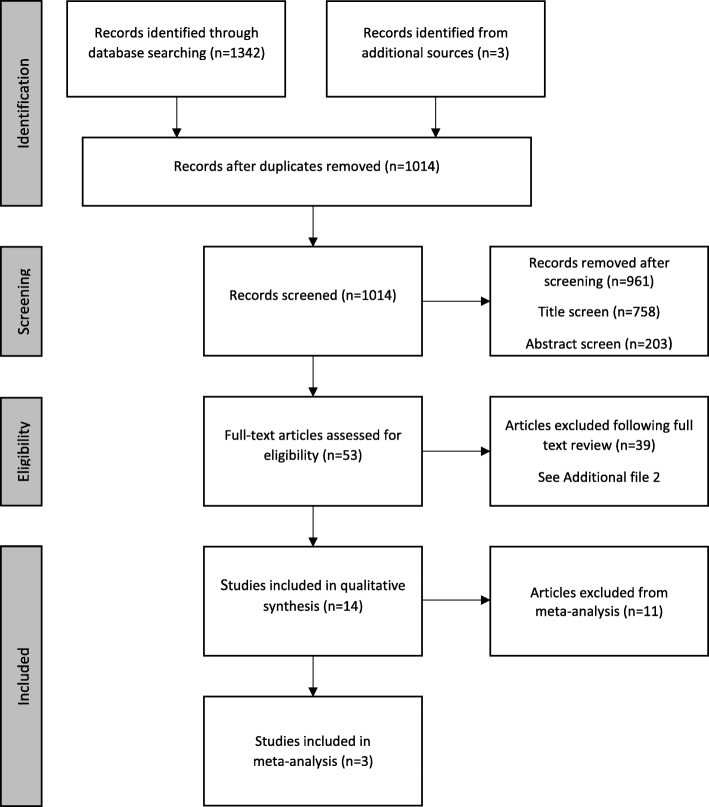
Summary of study selection process based on PRISMA flow diagram [49]

Following removal of duplicates, we identified 1014 articles through databases searches, reference lists and citing articles. The results for each database searched can found in Additional file 1. Title and abstract screening identified 53 articles for full text review of which 39 did not fulfil selection criteria for data analysis (See Additional file 2 for details). Fourteen studies were included in qualitative data synthesis and three were included in quantitative data synthesis. Figure 2 summarises the search results.

The 14 studies included a total of 257 patients with idiopathic pulmonary fibrosis. There was significant heterogeneity within the included studies in terms of study design, breath analysis technique and biomarkers investigated. Sixty-seven targeted biomarkers were measured with additional non-targeted metabolomic studies. Table 1 summarises the results of the included studies with reference to the primary and secondary research questions. None of the studies included in this review made comparison between IPF patients with evidence of honeycombing and those without documented honeycombing.

**Table 1:** Summary of characteristics and results of all included studies with reference to primary and secondary review questions. ^a^Decliners defined as drop on FVC of ≥10% or D_LCO_ ≥ 15% in preceding 6–12 months. All studies were case-control study design except for Ono (2008 and Kotecha (2016) which were uncontrolled cohort studies

Reference	Country	Biomarkers of interest	Sample Medium	Analytical method	IPF (n)	Healthy controls (n)	Primary Question – IPF vs healthy controls	Secondary Question – Lung function correlation	Secondary Question – treated vs untreated
[58]	Greece	8-isoprostane, H_2_0_2_	EBC	EIA, Enzyme assay	16	15	8-isoprostane and H_2_0_2_ higher in IPF group.	Negative correlation with D_LCO_% for both H_2_0_2_ and 8-isoprostane.	Not reported
[65]	USA	Non-targeted VOCs	Exhaled breath	Colorimetric sensing	15	21	Sensitivity for IPF group 40.0%, Specificity 92.3%.	Not reported	Not reported
[67]	Japan	CysLTs, FeNO_50_	EBC, Exhaled breath	EIA, Online chemiluminescence analyser	14	0	Not reported	No correlation between CysLTs and lung function parameters.	Not reported
[59]	Italy	Metallic elements	EBC	ICP-MS	19	33	Ni, Cr, Si higher in IPF group. Co, Fe, Cu, Se, Mo lower in IPF group.	No correlation between metallic elements and lung function parameters	No difference in metallic elements based on treatment received
[60]	Czech	Nitrite, Nitrate	EBC	LC with fluorescence detection	13	29	Nitrite levels higher in IPF group. Nitrate levels lower in IPF group	Not reported	Not reported
[55]	Japan	FeNO_50_, C_alv_NO, J_aw_NO, 42 Cytokines	Exhaled breath EBC	Online chemiluminescence analyser, Human cytokine antibody assay	13	10	No difference in FeNO_50_, C_alv_NO, J_aw_NO or cytokines.	Not reported	Not reported
[61]	Italy	MDA	EBC	HPLC with fluorescence detection	38	14	No difference in MDA levels.	Not reported	Not reported
[56]	China	C_alv_NO, J_aw_NO	Exhaled breath	Online chemiluminescence analyser	14	12	C_alv_NO higher in IPF group. No difference in J_aw_NO.	Not reported	Not reported
[62]	Japan	8-isoprostane	EBC	EIA	6	6	8-isoprostane levels higher in IPF group.	Not reported	Not reported
[63]	USA	Total LPA and sub-species	EBC	LC-MS	11	11	No difference in Total LPA. 22:4 LPA higher in IPF group.	No correlation between 22:4 LPA and lung function parameters. Total LPA not reported	Not reported
[57]	Italy	FeNO_50_, FeNO_100_, FeNO_150_, C_alv_NO	Exhaled breath	Online electrochemical analyser	32	30	FeNO_50–150_ and C_alv_NO higher in the IPF group.	Not reported	Not reported
[68]	UK	C_alv_NO	Exhaled breath	Online chemiluminescence analyser	27 [Stable (*n* = 16) vs Decliners^a^(*n* = 9)]	0	Not reported	C_alv_NO higher in decliners than stable patients.	No difference in C_alv_NO in treated vs untreated groups.
[64]	Switzerland	Non-targeted metabolomics	EBC	UHPLC-HRMS	10	10	One consistent discriminative metabolite (unidentifiable). Higher in IPF patients than controls.	Not reported	Not reported
[66]	Japan	Non-targeted VOCs	Exhaled breath	MCC-IMS	40	55	5 discriminative VOCs; p-cymene, acetoin, isoprene, ethylbenzene and an unidentified VOC. P-cymene was lower in IPF while the rest are higher.	P-cymene showed negative correlation with VC, %VC, FVC, %FVC, D_LCO_, %D_LCO_.	Not reported

*IPF* idiopathic pulmonary fibrosis, *H*_*2*_*0*_*2*_ hydrogen peroxide, *EBC* exhaled breath condensate, *EIA* enzyme immunoassay, *D*_*LCO*_ diffusion capacity for carbon monxide, *D*_*LCO*_ *%* diffusion capacity for carbon monoxide % predicted, *VOC* volatile organic compound, *CysLT* cysteinyl leukotriene, FeNO_50/100/150_, fractionated exhaled nitric oxide at 50 ml/100 ml/150 ml per second, C_alv_NO, alveolar nitric oxide concentration; J_aw_NO, airway flux of nitric oxide; *ICP* inductively coupled plasma, *MS* mass spectrometry, *Ni* nickel, *Cr* chromium, *Si* silicon, *Co* cobalt, *Fe* iron, *Cu* copper, *Se* selenium, *Mo* molybdenum, *LC* liquid chromatography, *MDA* malondialdehyde, *HPLC* high performance liquid chromatography, *LPA* lysophosphatidic acid, *22:4 LPA* docosatraenoyl lysophosphatidic acid, *UHPLC* ultra-high performance liquid chromatography, *HRMS* high resolution mass spectrometry, *MCC* multi-capillary column, *IMS* ion mobility spectrometry, *VC* vital capacity, *VC %* vital capacity % predicted, *FVC* forced vital capacity, *FVC %* forced viral capacity % predicted

### Study quality and risk of bias

**Fig. 3:**
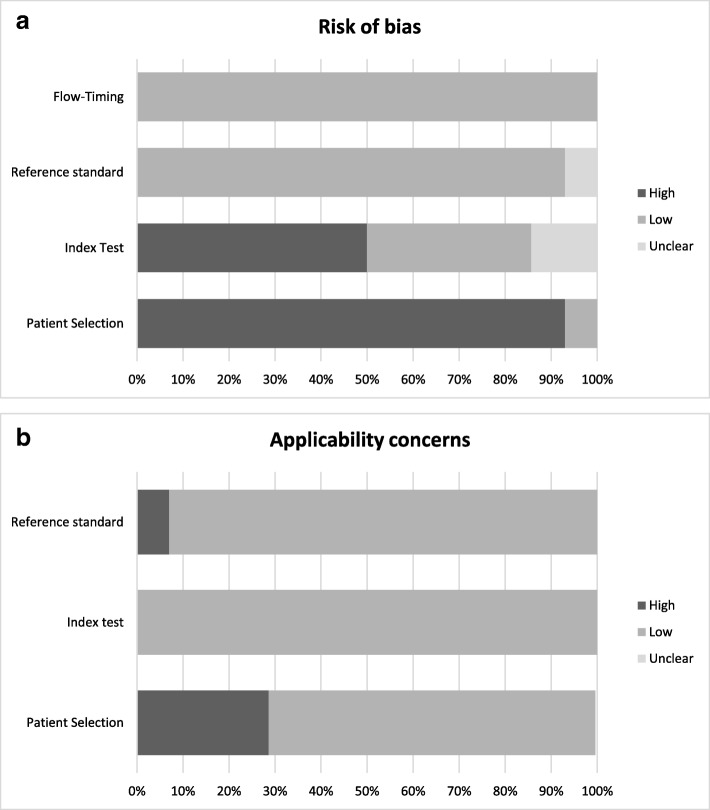
Proportion of studies included with high, low or unclear risk of bias (**a**) and applicability concerns (**b**) as per QUADAS-2 tool

Figure 3 summarises the risk of bias and applicability concern across the domains of the QUADAS-2 tool. The quality assessment for each study using the QUADAS-2 tool is presented in Additional file 3. In general, there were significant issues with bias particularly concerning patient selection. The majority of the studies were of a case-control design, however controls were frequently not matched for age and smoking status, both of which have been shown to influence results of breath analysis [53]. Sample sizes were small across the spectrum of studies included with the number of IPF patients ranging from 6 to 40. No power calculations were reported in any of the studies. Tests of breath biomarkers are prone to contamination from several sources including environmental, nasal and oral [53]. There was wide variation on how these were controlled, and the degree of methodological detail provided. There was also variation in the analytical equipment and/or assays used, which has also been shown to influence results and makes comparison between studies more difficult [39, 53, 54].

### Qualitative data synthesis

#### Which breath biomarkers are significantly different in the IPF population compared to healthy controls?

Twelve studies compared breath biomarkers in IPF compared to healthy controls. Twenty biomarkers were found to discriminate between IPF patients and healthy controls. These are listed in Table 2.

**Table 2:** Biomarkers reported to discriminate between IPF patients and healthy controls. Direction of discrimination and reported *p*-value. ^a^C_alv_NO. ^b^FeNO_50_/FeNO_100_/FeNO_150_/C_alv_NO

Biomarker	Sample Medium	Discrimination	*p*-value	References
Nitric Oxide	Exhaled breath	Higher in IPF	0.0001, < 0.0001	[62]^a^, [65]^b^
8-isoprostane	EBC	Higher in IPF	0.02, < 0.05	[58], [62]
Hydrogen Peroxide	EBC	Higher in IPF	0.003	[58]
Nickel	EBC	Higher in IPF	< 0.05	[59]
Chromium	EBC	Higher in IPF	< 0.05	
Silicon	EBC	Higher in IPF	< 0.05	
Cobalt	EBC	Lower in IPF	< 0.05	
Iron	EBC	Lower in IPF	< 0.05	
Copper	EBC	Lower in IPF	< 0.05	
Selenium	EBC	Lower in IPF	< 0.05	
Molybdenum	EBC	Lower in IPF	< 0.05	
Nitrite	EBC	Higher in IPF	< 0.01	[60]
Nitrate	EBC	Lower in IPF	< 0.01	
22:4 LPA	EBC	Higher in IPF	0.001	[63]
Unidentifiable metabolite	EBC	Higher in IPF	≤0.01	[64]
p-cymene	Exhaled breath	Lower in IPF	< 0.001	[66]
Acetoin	Exhaled breath	Higher in IPF	< 0.001	
Isoprene	Exhaled breath	Higher in IPF	< 0.001	
Ethylbenzene	Exhaled breath	Higher in IPF	< 0.001	
Unidentified VOC	Exhaled breath	Higher in IPF	< 0.001	

*IPF* idiopathic pulmonary fibrosis, *EBC* exhaled breath condensate, *22:4 LPA* Docosatetraenoyl lypophosphatidic acid, *VOC* volatile organic compound, *C*_*alv*_*NO* alveolar nitric oxide concentration, *FeNO*_*50/100/150*_ fractionated exhaled nitric oxide at 50 ml/100 ml/150 ml per second

Exhaled nitric oxide was measured in three studies; [55–57] two studies reported both FeNO_50_ and C_alv_NO [55, 57], and a third reported solely C_alv_NO. [56] Furukawa et al [55] found no difference in FeNO_50_ levels in IPF patients compared to controls, although the two groups were not well matched for gender or smoking history. Cameli et al [57] found that FeNO levels were higher in patients with IPF at flow rates of 50 ml/s as well as 100 ml/s (FeNO_100_) and 150 ml/s (FeNO_150_). These two studies, along with a study by Zhao et al, [56] also reported C_alv_NO in patients with IPF and healthy controls (see quantitative data synthesis).

Eight studies measured biomarkers present in EBC in patients with IPF and healthy controls [55, 58–64]. These included markers of oxidative stress [8-isoprostane, hydrogen peroxide (H_2_0_2_), malondialdehyde (MDA)], markers of nitrosative stress (nitrite and nitrate), metallic elements, and cytokines. Two studies found significantly higher levels of 8-isoprostone in IPF patients (25-74 pg/mL) compared to controls (5-33 pg/mL) [58, 62]. One study investigated lysophosphatidic acid (LPA) species in EBC from 11 IPF patients and 11 healthy controls using liquid chromatography-tandem mass spectrometry to identify the presence of total LPA and subspecies of LPA [63]. There was no difference in total LPA levels between the two groups but one species, docosatetraenoyl (22:4) LPA, was significantly higher in IPF compared to controls. These results may have been skewed by an outlier patient with extremely high levels of 22:4 LPA. This patient was subsequently hospitalised and treated for an exacerbation of IPF within ten days of collection and received urgent lung transplantation. There were no data presented that excluded this patient. Rindlisbacher et al [64] analysed EBC samples from ten patients with a diagnosis of IPF and ten age-matched controls using ultra high-performance liquid chromatography coupled to high-resolution mass spectrometry to identify discriminative metabolites using a non-targeted approach. A validation set of samples was taken from eight of the IPF patients and eight additional controls. The initial analysis identified 58 metabolic features which were significantly different between the IPF group and the healthy controls with 48 being identified in the validation set. Only two discriminative features were found to be present in both the pilot and the validation sets and of these only one was regulated in the same direction in both sets. This discriminative feature was two-fold up-regulated in IPF compared to healthy controls. They speculated that the potential molecular formula was C_21_H_44_N_2_O but were unable to identify a metabolite from known databases.

Two studies assessed VOCs in IPF patients and healthy controls [65, 66]. Yamada et al [66] examined VOCs in breath samples of 40 patients with IPF and 55 healthy controls. Samples were analysed using multicapillary column ion mobility spectrometry. Through this method they identified 85 VOC peaks in IPF patients and healthy controls, with significant differences in five. They identified these as p-cymene, acetoin, isoprene, ethylbenzene and an unidentified compound (peak 67). P-cymene was found to be lower in patients with IPF while the other compounds were higher in IPF. Isoprene was found to carry the highest diagnostic accuracy. This study was limited by the age difference between the two groups (IPF mean age 70, control mean age 38) and gender differences with a higher proportion of females in the control group. Another study assessed the ability of a colorimetric sensor to detect patterns of VOCs in lung cancer patients [65]. They also included patients with IPF and healthy controls in the cohort. Sensitivity of 40% and specificity of 93% for IPF was reported although this was not validated. Diagnosis was confirmed either clinically or histologically, however international guidelines were not referenced.

#### Do levels of breath biomarkers in the IPF population correlate with lung function parameters?

Six studies assessed correlation between breath biomarkers and lung function in IPF patients [58, 59, 63, 66–68]. Kotecha et al [68] compared C_alv_NO levels in 27 IPF patients whose lung function had been declining in the previous 6–12 months to those with stable lung function. C_alv_NO levels were noted to be significantly higher in the 16 patients with declining lung function. Psathakis et al [58] found a negative correlation with D_LCO_ % predicted with both H_2_0_2_ and 8-isoprostane although no association was noted with other measures of pulmonary function. Yamada et al [66] found that P-cymene had a negative correlation with lung function parameters including FVC and D_LCO_. Ethylbenzene had a negative correlation with %D_LCO_/VA (diffusion capacity per litre lung volume). No correlation was noted with levels of cysteinyl leukotrienes (CysLTs), Docosatetraenoyl (22:4) LPA or metallic elements in EBC and lung function parameters in IPF [59, 63, 67].

#### Do levels of breath biomarkers in the IPF population differ between treated and untreated patients?

Two studies compared breath biomarkers in IPF patients receiving treatment. Kotecha et al [68], found no difference in C_alv_NO levels in IPF patients receiving treatment with immunosuppression (either corticosteroids alone or in combination with azathioprine) and untreated patients. Corradi et al [59] found that there was no treatment effect on the presence of metallic elements in EBC of IPF patients. Treatment regimens included steroids, immunosuppression and pirfenidone, although this group of patients included both IPF and non-specific interstitial pneumonia (NSIP) diagnoses, and only two patients were receiving pirfenidone.

### Quantitative data synthesis

**Fig. 4:**
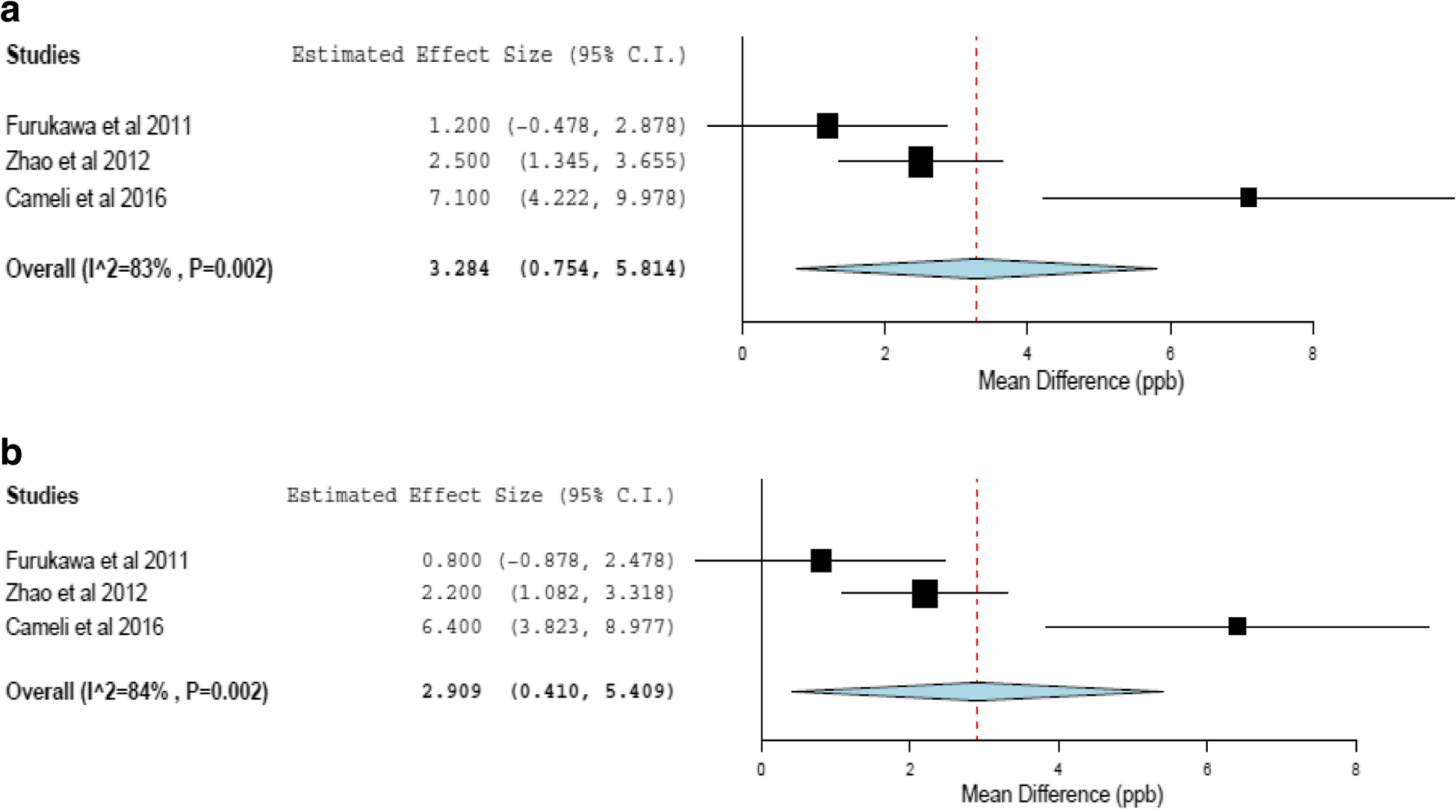
Random effects meta-analysis of mean difference in CalvNO (ppb) between groups (IPF vs Healthy controls) using Tsoukias and George (**a**) and Condorelli (**b**) methods of calculation

Three studies compared C_alv_NO levels in IPF patients and controls and were included in meta-analysis [55–57]. Each study reported C_alv_NO using both the Tsoukias and George method [36] and corrected for axial diffusion using the Condorelli method [69]. The range of values reported for C_alv_NO using the Tsoukias and George method was 4.4–11.8 ppb for IPF patients and 2.9–5.1 ppb for healthy controls. Using the Condorelli method the range was 3.9–10. 7 ppb for IPF patients and 1.7–4.5 ppb for healthy controls. We performed meta-analysis to compare mean difference in C_alv_NO (ppb) levels between IPF patients and healthy controls, using both Tsoukias and George and Condorelli methods. Figure 4 shows the forest plots. Meta-analysis suggested that C_alv_NO was significantly higher in patients with IPF using either method, however the total number of patients included was small (*n* = 101) and significant heterogeneity between the studies was noted (I^2^ = 83% & 84%, *p* = 0.002).

## Discussion

This systematic review has highlighted the heterogeneity of results from breath research in IPF. Over 60 known biomarkers and multiple unknown molecules have been investigated within a total patient cohort of less than 300 patients using a wide variety of analytical techniques. In this context, it is difficult to draw any firm conclusions regarding potentially clinically relevant markers. Twenty individual biomarkers were reported to discriminate between IPF and healthy controls however most of the studies did not include a validation cohort and there was an absence of longitudinal data. Only two of these biomarkers, exhaled NO and 8-isoprostane, have been studied more than once.

Exhaled NO is a breath biomarker which is easily measurable and already used in asthma care [32–35]. NO is a free radical that is implicated in a wide variety of physiological and pathophysiological processes [70]. It has been suggested that NO may increase the expression of pro-fibrotic mediators, such as transforming growth factor-β (TGF-β1), in lung fibrosis [71]. Two studies reported that measurements of exhaled nitric oxide were elevated in IPF compared to healthy controls [56, 57]. This is supported by an additional study which found that exhaled NO levels measured at a non-specific flow rate between 5 and 6 L/min were significantly higher in patients with a diagnosis of cryptogenic fibrosing alveolitis (CFA) compared to healthy non-smoking controls [72]. This study was excluded from this systematic review as it preceded international standards for IPF diagnosis. In clinical practice FeNO_50_ is the most common method used for measuring and reporting NO in breath. NO measured at this flow rate contains a higher proportion of airway and lower proportion of alveolar NO [54]. FeNO_50_ did not show consistency in IPF, [55, 57] or other forms of interstitial lung disease [73, 74]. FeNO_50_ levels in IPF patients ranged from 20.8–31.5 ppb [55, 57, 67]. It has been reported that the upper limit of FeNO_50_ in healthy adults ranges from 30.3–50.6 ppb depending on age and gender [75].

Although, FeNO_50_ is unlikely to be of use in IPF, C_alv_NO may have potential as a clinical biomarker. Meta-analysis in this review, while limited by small sample size and significant heterogeneity, suggests that C_alv_NO is higher in IPF patients compared to controls. Average C_alv_NO levels for patients with IPF were 8.5 (±5.5) ppb compared to 4.4 (±2.2) ppb for healthy controls. In a recent study of 433 healthy individuals, the upper limit of C_alv_NO reported was 3.88-3.93, depending on age [76]. Elevated alveolar NO supports a pathophysiological role in lung fibrosis. Nitrite, another mediator of nitrosative stress was also elevated in pulmonary fibrosis [60, 77]. C_alv_NO, but not FeNO, has also been reported to be elevated in ILD related to systemic sclerosis [78–80]. Increased exhaled NO levels have been described in other forms of ILD including hypersensitivity pneumonitis (HP), asbestosis and connective tissue disease related ILD (CTD-ILD), limiting the potential of the test as a discriminative diagnostic tool [73, 74, 81–85]. An alternative role for C_alv_NO may be in disease monitoring. Kotecha et al [68] suggested that C_alv_NO levels were reflective of deteriorating lung function. This is consistent with other studies suggesting that C_alv_NO levels are inversely related to lung function parameters in fibrotic ILD, a phenomenon not seen with FeNO [73, 81, 83]. However, a paucity of longitudinal data makes it difficult to draw any firm conclusions. Likewise, there is insufficient evidence to suggest a use for NO in monitoring treatment response in IPF. Only one study reported on treatment effect and found no difference in C_alv_NO levels between patients treated with immunosuppressive therapy and untreated patients [68]. Lower levels of exhaled NO were noted in patients with CFA receiving treatment with oral corticosteroids [72]. In both studies patients were receiving either steroid monotherapy or combination with azathioprine, a treatment regimen which has since been shown to have negative outcomes in IPF and is no longer recommended [86]. No studies have examined exhaled NO levels in patients taking antifibrotic therapy, the treatment of choice for IPF.

Several studies investigated biomarkers in EBC implicated in the pathogenesis of IPF [55, 58–64, 67]. Oxidative stress is one potential mechanism suggested in the fibrotic process [87]. In vivo, a variety of markers have been used as a surrogate index of oxidative stress [31]. A recent systematic review published in *Respiratory Research* has identified all the relevant markers of oxidative stress investigated in IPF [88]. These include H_2_0_2_, a reactive oxygen species, and 8-isoprostane, a prostaglandin-like compound formed by lipid peroxidation. Both were found to be elevated in EBC in patients with IPF [58, 62]. 8-isoprostane has also been reported to be elevated in other forms of fibrotic ILD. [82, 89] MDA, another marker of lipid peroxidation, was not elevated in EBC of IPF patients compared the healthy controls [61]. However, the authors speculated that this may have been reflective of a treatment effect as the majority of the IPF patients were receiving either oral corticosteroids or N-acetylcysteine. Additional markers of oxidative stress such as ethane have been reported to be higher in exhaled breath of patients with IPF and other forms of ILD [90].

Another study reported differences in concentration of metallic elements in EBC of patients with IPF compared to controls [59]. Metal workers are overrepresented in the IPF population and there is speculation that metal dust may be implicated in the disease process [91, 92]. The authors identified that nickel, chromium and silicon were present in higher concentrations in IPF patients and cobalt, iron, copper, selenium and molybdenum were higher in controls. This is inconsistent with a previously reported study using bronchoalveolar lavage fluid (BALF) which showed lower levels of chromium in IPF compared to controls and higher levels of iron [93].

Cytokines, such as tumour necrosis factor-α (TNF-α) and TGF-β, have been strongly implicated in cell signalling in IPF [94, 95]. However no significant differences were noted in levels of 42 cytokines in EBC of patients with IPF compared to controls [55]. LPA, a bioactive lysophospholipid which is believed to have a role in fibroblast migration, has been shown to be elevated in BALF of IPF patients [96]. One study did identify elevated levels of 22:4 LPA, a single subspecies of LPA, in IPF EBC [63], however total LPA levels were not significantly different. A recent phase II trial of a selective inhibitor of autotaxin (GLPG1690), a key enzyme in the production of LPA, produced promising safety and efficacy results as well as targeted reduction in plasma concentrations of another subspecies of LPA, C18:2 [97]. In the breath study, mean EBC levels of 18:2 LPA were higher IPF patients compared to controls, although not significantly [63], however studies are warranted to assess potential as a prognostic and therapeutic biomarker.

The evidence for EBC biomarkers in IPF is limited. These were primarily exploratory studies involving small cohorts and apart from 8-isoprostane, have not been reproduced. Currently a biomarker with diagnostic potential has not been identified. There is no evidence that EBC biomarkers distinguish between IPF and other forms of fibrotic ILD. Likewise, evidence is limited regarding their value as a prognostic marker. Levels of 8-isoprostane and H_2_O_2_ did appear to have a negative correlation with D_LCO_ % predicted in IPF but no relationship with other lung function parameters [58]. Another study suggested no correlation between in EBC H_2_O_2_ and lung function in 21 patients with mixed ILD including eight patients with IPF [98]. EBC is an appealing method of breath analysis, particularly as a liquid sample is obtained allowing a variety of analytical methods to be performed including specific assays, metabolomic and proteomic techniques. One possible disadvantage to EBC measurement in interstitial lung diseases, is that the technique predominantly samples respiratory lining fluid and may not accurately reflect a disease process in the lung interstitium. 8-isoprostane and H_2_O_2_ have both been reported to be elevated in airways disease [99–103], while MDA was shown to be higher in patients with asthma, COPD and bronchiectasis compared to controls [61]. H_2_O_2_ levels have been reported to be lower in patients with mixed ILD compared to obstructive lung disease [104]. Identification of molecules with a high specificity for interstitial fibrosis may be challenging using EBC as they are likely to be present in extremely small concentrations. One metabolomic study in EBC identified a novel metabolite which could consistently discriminate between IPF and healthy controls [64]. Unfortunately in this small pilot study, the metabolite could not be identified but this work shows promise and suggests that further work in this field is warranted.

VOCs offer a promising alternative source of breath biomarkers in IPF. The advantage of breath volatiles is that they could potentially travel from the blood or interstitium unaltered before detection in exhaled breath and therefore contain metabolites with high specificity for the fibrotic process. VOC work has been limited in IPF to date. Yamada et al [66] undertook a breathomic study using multicapillary column ion mobility spectrometry. They identified five compounds which could discriminate between IPF and healthy controls to varying degrees of accuracy. Isoprene carried the highest diagnostic accuracy with an area under the receiver operative characteristic curve (AUROC) of 0.81. Isoprene is a by-product of normal cholesterol synthesis and elevated levels have been noted a variety of disease states [105, 106], and therefore may be of limited use as a specific biomarker in IPF. Interestingly, lower levels of exhaled isoprene have been reported in advanced fibrosis of the liver [107]. P-cymene, which demonstrated an AUROC of 0.80, may show the most promise as a clinical biomarker as it displayed the strongest correlation with markers of disease severity. P-cymene is thought to have anti-oxidant properties and its reduction in IPF may correlate with the putative theory of increased oxidative stress [108]. This was the largest of the included studies with 95 subjects recruited. Despite this there were issues with case-control matching as the control group were significantly younger than the IPF group and were also not matched for gender and smoking history. However, this study highlights the potential for breathomic discovery of novel biomarker with both diagnostic and prognostic potential. Once specific biomarkers or patterns of biomarkers (“breathprints”) have been identified, tailored diagnostic tools such as electronic noses which are more accessible in the clinical setting, could be developed. “E-noses” capable of detecting VOCs have been successfully used to phenotype and monitor treatment response in a number of conditions including COPD and obstructive sleep apnoea [109–111].

The process of biomarker development is often described as the “biomarker pipeline” and thus far none of the potential candidates have progressed past the biomarker discovery phase [112]. It can take decades to translate from the discovery phase to a clinically validated biomarker and the results of this systematic review likely reflect the fact that biomarker development in IPF remains in its infancy. Studies of blood biomarkers in IPF, in which there has been a larger body of work, have thus far failed to identify a candidate with sufficient diagnostic or prognostic accuracy [5, 113]. The pathophysiology of IPF is complex and our understanding of the multiple processes and pathways is evolving [114]. This is a double edged sword as multiple potential biomarker candidates may exist, but identifying an individual marker which independently correlates with disease activity is extremely challenging. Likewise, development of biomarkers in IPF is particularly difficult due to lack of a gold standard diagnostic test. Diagnosis is made on consensus from a combination of available information, predominantly radiological, and it is not uncommon for this to be a “working diagnosis” [115, 116]. Uncertainty of the accuracy of the reference standard inherently limits the reliability of the potential biomarker. It would be unfeasible to perform a biomarker study solely in patients with biopsy proven disease, as these are performed infrequently and generally in a younger, non-representative cohort [8]. VOCs have been measured in the “headspace” of in-vitro models of disease, for example lung cancer cell lines [117], however it is difficult to replicate IPF in cellular models [118].

The hope is that biomarker discovery studies inform our understanding of the pathophysiology of IPF and improve accuracy of diagnosis. In addition, novel biomarkers may be able distinguish some of the phenotypes that appear to exist within IPF [10]. Progress is certainly required if a clinically relevant breath biomarker is to emerge in IPF. Further breathomics studies are warranted. Longitudinal studies would be beneficial as this would increase the data pool considerably and allow individual reproducibility to be assessed. It would also provide the opportunity to better evaluate the impact of initiating treatment on biomarker levels, an issue which may confound cross-sectional studies.

### Limitations

There are several limitations to this systematic review. We defined IPF based on international consensus guidelines [2, 50]. This necessitated excluding studies involving patients that did not meet these diagnostic standards. One of these studies included patients with a diagnosis of CFA, pre-dating diagnostic guidelines [72]. This may have included patients with IPF, but also those which would now be classified as NSIP. Several other studies included patients with IPF as a composite with other forms of fibrotic ILD such as NSIP, [83] CTD-ILD, [82] or HP [81]. Two studies measured breath biomarkers in IPF but made comparison with other forms of ILD rather than healthy controls [73, 90]. This was outside the remit of this systematic review. We excluded studies that were not published in English. The search strategy identified two studies, one published in German and one in Polish, which otherwise met criteria for full text review [119, 120]. We excluded studies presented in abstract form as insufficient detail was provided to allow accurate and comprehensive data extraction and quality assessment. These included some studies which appeared to otherwise fulfil inclusion criteria (see Additional file 2 for details).

## Conclusions

In this PRISMA-compliant systematic review we identified a heterogeneous group of studies investigating a wide variety of breath biomarkers in IPF using a range of analytical techniques. Evidence for the use of specific biomarkers was inconclusive. Twenty individual biomarkers were reported to discriminate between IPF and healthy controls. Meta-analysis of three studies indicated that C_alv_NO levels are significantly higher in patients with IPF compared to healthy controls. C_alv_NO, 8-isoprostone, hydrogen peroxide and p-cymene demonstrated correlation with lung function parameters in IPF patients. There was a lack of studies investigating relevant treatment options in IPF. None of the studies compared breath biomarkers in patients with IPF in relation to the presence of honeycombing. Recent studies have indicated that a “breathomic” approach may identify biomarkers with specificity for IPF [64, 66]. This may be of significant clinical benefit as diagnosis may be challenging due to difficulties distinguishing between IPF and other fibrotic lung diseases, particularly as a large proportion of patients are not suitable for surgical lung biopsy. Larger studies with longitudinal data are required to identify biomarkers with diagnostic and prognostic potential.

## Additional files

Additional file 1: Search results per database. (DOCX 12 kb)

Additional file 2: Studies excluded following full text review (DOCX 18 kb)

Additional file 3: Summary of QUADAS-2 assessment for each study. (DOCX 15 kb)

## Abbreviations

22:4 LPA: Docosatraenoyl lysophosphatidic acid
AUROC: Area under the receiver operative characteristic curve
BALF: Bronchoalveolar lavage fluid
C: Carbon
C_alv_NO: Alveolar nitric oxide
C_aw_NO: Airway wall nitric oxide
CFA: Cryptogenic fibrosing alveolitis
Co: Cobalt
COPD: Chronic obstructive pulmonary disease
Cr: Chromium
CTD: Connective tissue disease
Cu: Copper
CysLT: Cysteinyl leukotriene
D_aw_NO: Diffusion capacity for nitric oxide
D_LCO_: Diffusion capacity for carbon monoxide
EBC: Exhaled breath condensate
EIA: Enzyme immunoassay
Fe: Iron
FeNO: Fractionated exhaled nitric oxide
FVC: Forced vital capacity
H: Hydrogen
H_2_O_2_: Hydrogen peroxide
HP: Hypersensitivity pneumonitis
HPLC: High performance liquid chromatography
HRMS: High resolution mass spectrometry
ICP: Inductively coupled plasma
ILD: Interstitial lung disease
IMS: Ion mobility spectrometry
IPF: Idiopathic pulmonary fibrosis
J_aw_NO: Airway flux of nitric oxide
KL-6: Krebs von den Lungen-6
LC: Liquid chromatography
LPA: Lysophosphatidic acid
MCC: Multi-capillary column
MDA: Malondialdehyde
MeSH: Medical subject headings
MMP7: Matrix metalloproteinase-7
Mo: Molybdenum
MS: Mass spectroscopy
N: Nitrogen
Ni: Nickel
NO: Nitric oxide
NSIP: Non-specific interstitial pneumonia
O: Oxygen
ppb: Part per billion
PRISMA: Preferred reporting items for systematic reviews and meta-analyses
PROFILE: Prospective observation of fibrosis in the lung clinical endpoints
QUADAS-2: Quality assessment tool for diagnostic accuracy studies
SD: Standard deviation
Se: Selenium
SE: Standard error
Si: Silicon
SP-A: Surfactant protein-A
SP-D: Surfactant protein-D
TGF-β: Transforming growth factor-β
TNF-α: Tumour necrosis factor-α
UHPLC: Ultra-high performance liquid chromatography
USA: United States of America
VA: Alveolar volume
VC: Vital capacity
VOC: Volatile organic compound
