# Supplementary material for: Breath biomarkers in idiopathic pulmonary fibrosis: a systematic review
Source: Respir Res. 2019 Jan 11;20:7. doi: 10.1186/s12931-019-0971-8 (PMC6329167; doi:10.1186/s12931-019-0971-8)
Supplement: Supplementary file 1 — Search results per database. (DOCX 12 kb) [file 12931_2019_971_MOESM1_ESM.docx]

| Search Number | Database | Date | Results | Results after duplicates removed |
| --- | --- | --- | --- | --- |
| 1 | MEDLINE (Pub med) | 8^th^ January 2018 | 349 | 349 |
| 2 | MEDLINE (Ovid) | 8^th^ January 2018 | 94 | 53 |
| 3 | EMBASE | 8^th^ January 2018 | 217 | 78 |
| 4 | Web of Science | 8^th^ January 2018 | 682 | 531 |
|  |  | TOTAL | 1342 | 1011 |

Appendix 1 – Search results per database.
