# Supplementary material for: Breath biomarkers in idiopathic pulmonary fibrosis: a systematic review
Source: Respir Res. 2019 Jan 11;20:7. doi: 10.1186/s12931-019-0971-8 (PMC6329167; doi:10.1186/s12931-019-0971-8)
Supplement: Supplementary file 2 — Studies excluded following full text review (DOCX 18 kb) [file 12931_2019_971_MOESM2_ESM.docx]

Appendix 2 – Studies excluded following full text review

| **Reference** | **Reason for exclusion** |
| --- | --- |
| Anhenn O, Rabis T, Sommerwerck U, Weinreich G, Baumbach JI, Kurth I, et al. Detection of differences in volatile organic compounds (VOCs) by ion mobility spectrometry (IMS) of exhaled breath in patients with interstitial lung diseases (ILDs) compared to healthy controls (HC). European Respiratory Journal Conference: European Respiratory Society Annual Congress. 2011;38. | Abstract only |
| Boots A, Schins R, Godschalk R, Albrecht C, Dallinga J, Bast A, et al. Volatile organic compounds may provide a new and promising tool for diagnosing interstitial lung diseases. European Respiratory Journal. 2013;42. | Abstract only |
| Boots AW, Schins RPF, Godschalk RWL, Albrecht C, Dallinga JW, Bast A, et al. The analysis of volatile organic compounds as a new and promising tool for diagnosing interstitial lung diseases. American Journal of Respiratory and Critical Care Medicine Conference: American Thoracic Society International Conference, ATS. 2012;185. | Abstract only |
| Brown LAS, Jones DP. Glutathione and Thiols. In: Roberts SM, Kehrer JP, Klotz L-O, editors. Studies on Experimental Toxicology and Pharmacology. Cham: Springer International Publishing; 2015. p. 131-47. | Not primary reaserch - review chapter |
| Cameli P, Bargagli E, Refini RM, Pieroni MG, Bennett D, Rottoli P. Exhaled nitric oxide in interstitial lung diseases. Respir Physiol Neurobiol. 2014;197:46-52. | Not IPF alone - composite of IPF and NSIP |
| Carpagnano GE, Lacedonia D, Soccio P, Caccavo I, Patricelli G, Foschino Barbaro MP. How strong is the association between IPF and lung cancer? An answer from airway's DNA. Medical Oncology. 2016;33 (11)(119). | Not biomarker - DNA |
| Carpagnano GE, Kharitonov SA, Wells AU, Pantelidis P, Du Bois RM, Barnes PJ. Increased vitronectin and endothelin-1 in the breath condensate of patients with fibrosing lung disease. Respiration. 2003;70(2):154-60. | Not IPF |
| Choi J. Exhaled nitric oxide in patients with intestitial lung disease: a pilot study. PhD dissertation. University of Pittsburg. 2008 | Unpublished (PhD thesis) |
| Chow S, Thomas PS, Malouf M, Yates DH. Exhaled breath condensate (EBC) biomarkers in pulmonary fibrosis. Journal of Breath Research. 2012;6(1) | Not IPF alone - composite of IPF and connective tissue related pulmonary fibrosis |
| Chow S, Yates DH, Thomas PS. Reproducibility of exhaled breath condensate markers. European Respiratory Journal. 2008;32(4):1124-6. | Not IPF alone - Pulmonary fibrosis not further characterised (does not meet diagnostic criteria for IPF) and research letter (not full test) |
| Ciarleglio G, Refini RM, Pieroni MG, Martino VA, Bargagli E, Rottoli P, et al. Exhaled carbon monoxide in sarcoidosis. Sarcoidosis, vasculitis, and diffuse lung diseases : official journal of WASOG. 2008;25(1):46-50. | Not IPF - composite of non-sarcoid ILD |
| Corradi M, Pignatti P, Brunetti G, Goldoni M, Caglieri A, Nava S, et al. Comparison between exhaled and bronchoalveolar lavage levels of hydrogen peroxide in patients with diffuse interstitial lung diseases. Acta Biomedica de l'Ateneo Parmense. 2008;79(SUPPL. 1):73-8. | No control group - comparison of breath and BAL |
| Fijten RRR, Smolinska A, Drent M, Dallinga JW, Mostard R, Pachen DM, et al. The necessity of external validation in exhaled breath research: a case study of sarcoidosis. J Breath Res. 2017;12(1):016004. | Not IPF |
| Fireman E, Shtark M, Priel IE, Shiner R, Mor R, Kivity S, et al. Hydrogen peroxide in exhaled breath condensate (EBC) vs eosinophil count in induced sputum (IS) in parenchymal vs airways lung diseases. Inflammation. 2007;30(1-2):44-51. | Not IPF |
| Folesani G, Corradi M, Goldoni M, Manini P, Acampa O, Andreoli R, et al. Urea in exhaled breath condensate of uraemics and patients with chronic airway diseases. Acta bio-medica : Atenei Parmensis. 2008;79 Suppl 1:79-86. | Not IPF |
| Gregus M, Foret F, Kindlova D, Pokojova E, Plutinsky M, Doubkova M, et al. Monitoring the ionic content of exhaled breath condensate in various respiratory diseases by capillary electrophoresis with contactless conductivity detection. Journal of Breath Research. 2015;9(2). | Not IPF alone - Pulmonary fibrosis not further characterised (does not meet diagnostic criteria for IPF) |
| Guilleminault L, Saint-Hilaire A, Favelle O, Caille A, Boissinot E, Henriet AC, et al. Can exhaled nitric oxide differentiate causes of pulmonary fibrosis? Respir Med. 2013;107(11):1789-96. | Inadequate control comparison - comparisons between IPF and non-IPF ILD and ILD vs Healthy controls |
| Guiot J, Henket M, Corhay J-L, Louis R. Serum IGFBP2 as a marker of idiopathic pulmonary fibrosis. European Respiratory Journal. 2015;46(suppl 59). | Abstract only |
| Hildebrand K, Krenke R, Przybylowski T, Fangrat A, Gorska K, Chazan R. [Influence of bronchoscopy on nitric oxide in exhaled air]. Pneumonologia i alergologia polska. 2006;74(1):26-31. | Non-English Language |
| Jackson AS, Sandrini A, Campbell C, Chow S, Thomas PS, Yates DH. Comparison of biomarkers in exhaled breath condensate and bronchoalveolar lavage. American journal of respiratory and critical care medicine. 2007;175(3):222-7. | Not IPF |
| Kanoh S, Kobayashi H, Motoyoshi K. Exhaled ethane: An in vivo biomarker of lipid peroxidation in interstitial lung diseases. Chest. 2005;128(4):2387-92. | Inadequate control comparison - comparisons between IPF and non-IPF fibrotic disease |
| Kuban P, Gregus M, Foret F, Skrickova J, Kindlova D, Doubkova M, et al. Monitoring of ionic content of exhaled breath condensate in various respiratory diseases by capillary electrophoresis with contactless conductivity detection. European Respiratory Journal Conference: European Respiratory Society Annual Congress. 2015;46. | Abstract only |
| Lehtimaki L, Kankaanranta H, Saarelainen S, Hahtola P, Jarvenpaa R, Koivula T, et al. Extended exhaled NO measurement differentiates between alveolar and bronchial inflammation. American journal of respiratory and critical care medicine. 2001;163(7):1557-61. | Not IPF alone - composite of IPF and HSP |
| Li F, Surolia R, Li H, Kulkarni T, Wang Z, Liu G, et al. Identification of pathogenic and prognostic anti-vimentin antibodies in idiopathic pulmonary fibrosis. American Journal of Respiratory and Critical Care Medicine Conference: American Thoracic Society International Conference, ATS. 2017;195. | Abstract only |
| Machado RF, Laskowski D, Deffenderfer O, Burch T, Zheng S, Mazzone PJ, et al. Detection of Lung Cancer by Sensor Array Analyses of Exhaled Breath. American journal of respiratory and critical care medicine. 2005;171(11):1286-91. | Not IPF |
| Montesi SB, Mathai SK, Brenner LN, Gorshkova I, Berdyshev E, Tager AM, et al. Docosatetraenyl Lysophosphatidic Acid (lpa) Is Elevated In Exhaled Breath Condensate In Idiopathic Pulmonary Fibrosis. American journal of respiratory and critical care medicine. 2014;189. | Abstract only |
| Montuschi P, Barnes PJ, Ciabattoni G. Measurement of 8-Isoprostane in Exhaled Breath Condensate. In: Armstrong D, editor. Advanced Protocols in Oxidative Stress Ii. Methods in Molecular Biology. 5942010. p. 73-84. | Not primary research - review chapter |
| Nagaraja C, Shashibhushan BL, Sagar, Asif M, Manjunath PH. Hydrogen peroxide in exhaled breath condensate: A clinical study. Lung India : official organ of Indian Chest Society. 2012;29(2):123-7. | Not IPF - ILD not further characterised. |
| Oishi K, Hirano T, Suetake R, Ohata S, Yamaji Y, Ito K, et al. Exhaled nitric oxide measurements in patients with acute-onset interstitial lung disease. J Breath Res. 2017;11(3):036001. | Not IPF |
| Ojanguren I, Cruz MJ, Villar A, Sanchez-Ortiz M, Morell F, Munoz X. Changes in PH in exhaled breath condensate after specific bronchial challenge test in patients with chronic hypersensitivity pneumonitis: a prospective study. BMC Pulm Med. 2015;15:109. | Not IPF |
| Paredi P, Kharitonov SA, Loukides S, Pantelidis P, du Bois RM, Barnes PJ. Exhaled nitric oxide is increased in active fibrosing alveolitis. Chest. 1999;115(5):1352-6. | Not IPF - diagnosis of cryptogenic fibrosing alveolitis. Diagnosis pre-dated guidelines for IPF diagnosis. |
| Riley MS, Porszasz J, Miranda J, Engelen MP, Brundage B, Wasserman K. Exhaled nitric oxide during exercise in primary pulmonary hypertension and pulmonary fibrosis. Chest. 1997;111(1):44-50. | Not IPF - composite of mixed fibrotic lung disease |
| Saleh D, Barnes PJ, Giaid A. Increased production of the potent oxidant peroxynitrite in the lungs of patients with idiopathic pulmonary fibrosis. American journal of respiratory and critical care medicine. 1997;155(5):1763-9. | Not breath |
| Shah K, Padilla ML, Dua S. A pilot study to measure 8-isoprostane levels in exhaled breath condensate of ambulatory patients with diffuse parenchymal lung disease. American Journal of Respiratory and Critical Care Medicine Conference: American Thoracic Society International Conference, ATS. 2012;185. | Abstract only |
| Schildge J. Nitric oxide in exhaled breath of patients with interstitial lung diseases. [German]. Pneumologie. 2011;65(3):143-8. | Non-English Language |
| Sinues P, Nussbaumer-Ochsner Y, Gaugg MT, Bregy L, Engler A, Zenobi R, et al. Exhaled breath analysis by real-time mass spectrometry in patients with pulmonary fibrosis. Chest. 2017;151(5):16A-A. | Abstract only |
| Strebel C, Rindlisbacher B, Guler S, Geiser T, Bovet C, Funke M. Exhaled breath condensate-a potential biomarker tool for patients with idiopathic pulmonary fibrosis? Qjm. 2016;109:S39. | Abstract only |
| Strebel C, Rindlisbacher B, Guler S, Geiser T, Bovet C, Funke M. Metabolite profile differences in exhaled breath condensate from patients with idiopathic pulmonary fibrosis compared to healthy individuals analyzed by mass spectrometry - A pilot study. Respiration. 2016;91 (5):420. | Abstract only |
| Szymanska E, Tinnevelt GH, Brodrick E, Williams M, Davies AN, van Manen HJ, et al. Increasing conclusiveness of clinical breath analysis by improved baseline correction of multi capillary column - ion mobility spectrometry (MCC-IMS) data. Journal of Pharmaceutical and Biomedical Analysis. 2016;127:170-5. | Not IPF alone - combined to make repiratory disease group |
