# Supplementary material for: Breath biomarkers in idiopathic pulmonary fibrosis: a systematic review
Source: Respir Res. 2019 Jan 11;20:7. doi: 10.1186/s12931-019-0971-8 (PMC6329167; doi:10.1186/s12931-019-0971-8)
Supplement: Supplementary file 3 — Summary of QUADAS-2 assessment for each study. (DOCX 15 kb) [file 12931_2019_971_MOESM3_ESM.docx]

| **Study** | **Domain 1 - Patient selection - bias** | **Domain 1 - Patient selection - applicability concerns** | **Domain 2 - Index test - bias** | **Domain 2 - Index test - applicability concerns** | **Domain 3 - Reference standard - bias** | **Domain 3 - Reference standard - applicability concerns** | **Domain 4 - Flow/Timing - bias** |
| --- | --- | --- | --- | --- | --- | --- | --- |
| Psathakis *et al* (2006) | High | Low | High | Low | Low | Low | Low |
| Mazzone *et al* (2007) | High | Low | Unclear | Low | Low | Low | Low |
| Ono *et al* (2008) | High | High | High | Low | Unclear | Low | Low |
| Corradi *et al* (2009) | High | Low | High | Low | Low | Low | Low |
| Rihak *et al* (2010) | High | High | High | Low | Low | High | Low |
| Bartoli *et al* (2011) | High | Low | High | Low | Low | Low | Low |
| Furukawa *et al* (2011) | High | Low | Low | Low | Low | Low | Low |
| Zhao *et al* (2012) | High | Low | Low | Low | Low | Low | Low |
| Montesi *et al* (2014) | High | High | High | Low | Low | Low | Low |
| Shimizu *et al* (2014) | High | High | High | Low | Low | Low | Low |
| Cameli *et al* (2016) | High | Low | Low | Low | Low | Low | Low |
| Kotecha *et al* (2016) | Low | Low | Low | Low | Low | Low | Low |
| Rindlisbacher *et al* (2017) | High | Low | Low | Low | Low | Low | Low |
| Yamada *et al* (2017) | High | Low | Unclear | Low | Low | Low | Low |

**Appendix 3** – Summary of QUADAS-2 assessment for each study.
